# Gastric Mucosa Fistula Secondary to Magnet Ingestion

**DOI:** 10.1097/PG9.0000000000000015

**Published:** 2020-11-04

**Authors:** Alyssa Lorenze, Syndey Downey, Lisa M. Costello, Cortney Ballengee Menchini

**Affiliations:** From the Department of Pediatrics, West Virginia University School of Medicine, Morgantown, WV.

## Abstract

A 15-month-old female was incidentally found to have foreign bodies in the left upper quadrant on chest and abdominal imaging. She had no witnessed ingestion or gastrointestinal symptoms. Subsequent esophagogastroduodenoscopy showed 11 magnets, which formed a gastric mucosa fistula making endoscopic removal difficult. This case highlights the dangers of high-powered magnets and the unique challenges they can pose to endoscopists.

## CASE REPORT

A previously healthy, well-grown 15-month-old female (8.7 kg) presented to the emergency department with several days of progressively worsening cough, congestion, and rhinorrhea.

**Figure 1.**
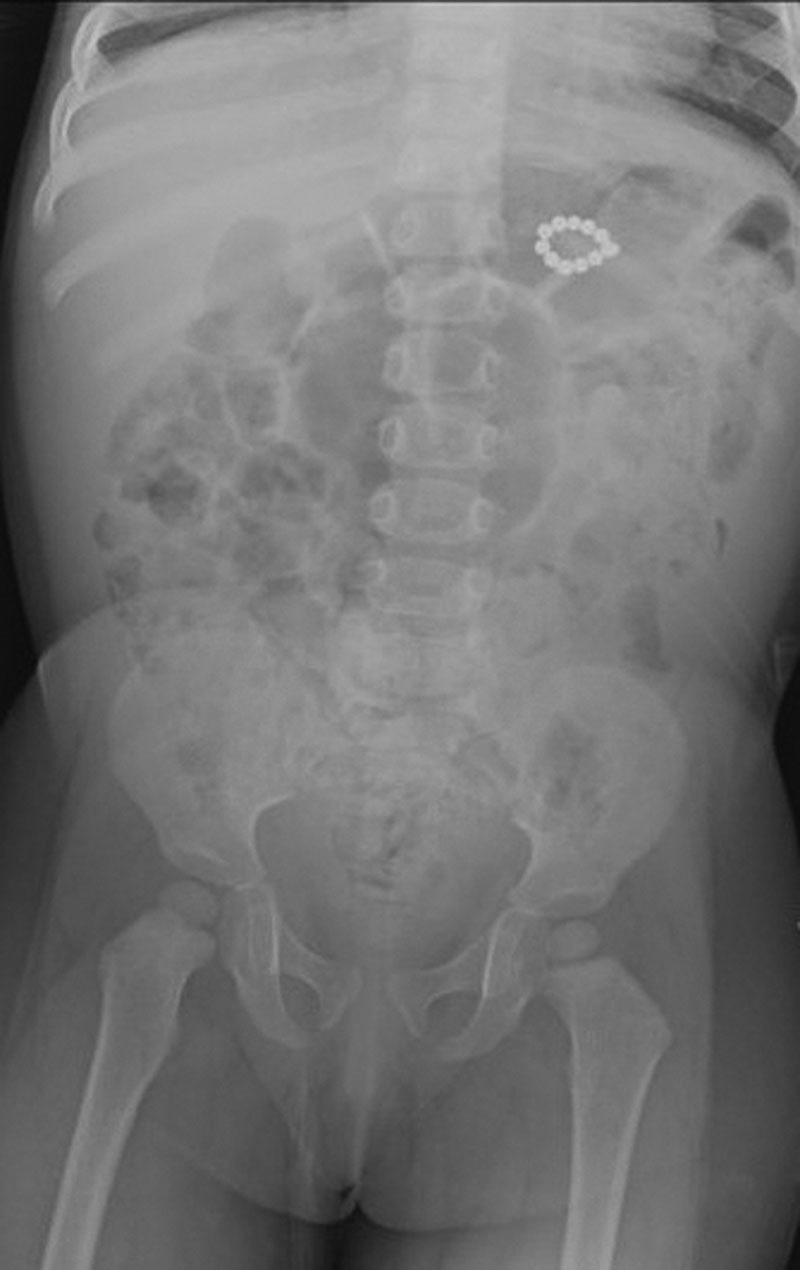
Abdominal X-ray.

**Figure 2.**
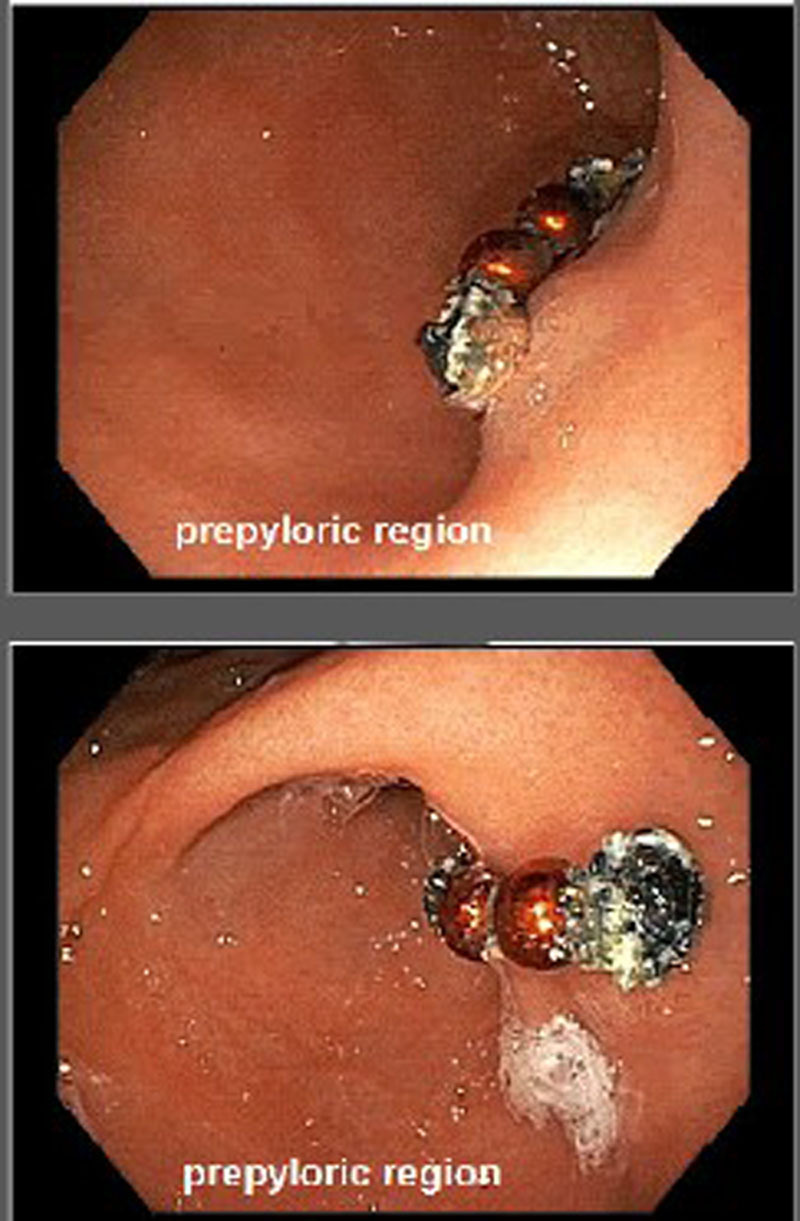
Endoscopic image, procedure 1.

Viral nasal swab for upper respiratory infection was positive for parainfluenza. Chest radiograph to evaluate for pneumonia was suggestive of swallowed foreign bodies. The parents did not report any witnessed ingestion. Abdominal radiograph confirmed a radiopaque beaded density over the gastric body (Fig. 1). The patient had no vomiting or abdominal pain. Esophagogastroduodenoscopy (EGD) using an Olympus gastroscope (GIF-H190) demonstrated a ring of 11 magnets pinching the gastric mucosa in the prepyloric region along the lesser curvature (Fig. 2). The mucosa appeared erythematous and congested at the contact site with the magnets. Multiple attempts at endoscopic removal with rat-toothed forceps and foreign body graspers were unsuccessful, and the procedure was aborted due to respiratory distress and concern for gastric perforation.

**Figure 3.**
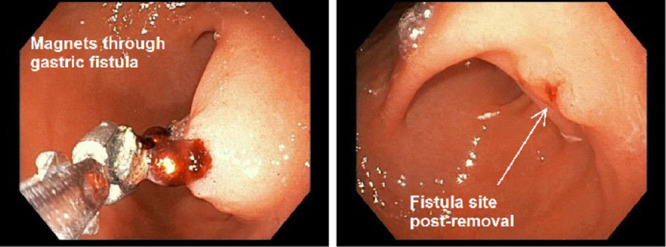
Endoscopic image, procedure 2.

Repeat EGD with possible exploratory laparotomy was postponed until resolution of the respiratory illness, during which the patient had no evidence of perforation on imaging. Six days later, a second EGD was performed with pediatric surgery available. The chain of magnets was still positioned in the prepyloric area of the stomach but with slightly less mucosal inflammation at the mucosal-magnet border (Fig. 3, left). The proximal magnet was broken from the chain using rat-toothed forceps and removed through the mouth. With reintroduction of the forceps, the next magnet was grasped, and the remaining magnets were easily pulled through the gastric fold in a linked chain. After the magnets were removed, a fistula was visualized (Fig. 3, right). When the area was irrigated, water could be seen entering the proximal stoma of the fistulous tract and exiting near the pylorus. Abdominal radiograph demonstrated that all foreign objects had been removed with no evidence of intraperitoneal free air or perforation.

Viewed postremoval, the magnets were less than 2.5 mm in diameter. The patient’s mother believed they came from a jewelry making kit in the home of a family member the patient had visited a week before hospital presentation. Following the procedure, the patient was admitted to the pediatric intensive care unit for close observation of respiratory status. The patient tolerated gradual reintroduction of oral feeds with daily upright and lateral imaging revealing no free air. She was discharged 2 days later, on hospital day 8, with no postoperative complications. Outpatient evaluation, 1 month later, revealed no symptoms and adequate weight gain. The case was reported to the Consumer Protection Safety Commission and assigned an investigator. However, a conclusion on the brand or manufacturer of the magnets was missing due to loss of contact with the family.

## DISCUSSION

Foreign body ingestions, specifically button batteries and high-powered magnets, remain one of the most challenging clinical scenarios faced by pediatric gastroenterologists. From 2002 to 2011, the incidence of magnetic ingestions in children rose 8.5 fold with >16,000 ingestions presenting to the emergency room (1). Ingestion of multiple magnets carries the risk for potential enteroenteric fistula formation, causing bowel ischemia/necrosis, perforation, and peritonitis (1, 2). Use of neodymium, or rare earth, in these magnets causes >5 times the attractive force of conventional magnets, leading to easier gastrointestinal injury and the need for prompt intervention. The North American Society for Pediatric Gastroenterology, Hepatology and Nutrition strongly lobbied for magnet restrictions in 2012, and in 2014 the United States Consumer Product Safety Commission passed federal standards effectively banning the sale of high-powered magnets (3). However, these regulations were overturned in appeals court, and further restrictions have not been enacted. A bill was introduced in the US Senate (S.3143—Magnet Injury Prevention Act) but remains in committee (4).

Indications and timing for intervention must take into consideration several factors, including patient size, number, and type of object(s) ingested, clinical symptoms, location, and time since ingestion (1). Children who ingest multiple magnets or a magnet and a metallic foreign body are more likely to require surgical intervention, thus leading many to proceed to endoscopic removal rather than observation alone (2). According to the North American Society for Pediatric Gastroenterology, Hepatology and Nutrition endoscopy committee, an asymptomatic patient who ingested either more than 1 magnet or a single magnet and a metallic object should undergo urgent endoscopic removal (in less than 24 hours) if the location is accessible (1). This is because patients may not become symptomatic until severe bowel injury has occurred (1). Our patient is unique because routine imaging for an upper respiratory illness led to the incidental discovery of multiple foreign bodies. Initial EGD showed a ring of magnets with inflamed surrounding mucosa, then repeat EGD 6 days later revealed a mature intragastric fistula. This highlights the ever-changing risks of retained gastrointestinal magnets. Only one other case of intragastric fistula has been reported in the literature (5). Because the time of the ingestion was unknown, and the patient had an underlying viral illness, the risk of anesthesia complications was higher. With variability in endoscopic management, it is important to have documented cases in the literature to aid in the complex medical decision-making to provide safe, prompt medical care. For unwitnessed, multiple magnet ingestions, it is advised to have pediatric surgery available, especially in the setting of a respiratory illness.
